# Factors associated with appendicular skeletal muscle mass among male Chinese patients with stable chronic obstructive pulmonary disease: A hospital-based cross-sectional study

**DOI:** 10.1097/MD.0000000000017361

**Published:** 2019-10-04

**Authors:** Yanxia Han, Zhenyun Wu, Yi Chen, Yanan Kan, Min Geng, Nuo Xu, Hongying Qian, Hai Fang Wang, Meie Niu

**Affiliations:** a Department of Respiratory Medicine, The First Affiliated Hospital of Soochow University; b School of Nursing, Soochow University, Suzhou, China.

**Keywords:** appendicular skeletal muscle mass, chronic obstructive pulmonary disease, fat-free mass, stable phase

## Abstract

Limbs muscle wasting is a common disorder in patients with chronic obstructive pulmonary disease (COPD) that limits daily activities and exercise intolerance, especially in males. The present study aimed to estimate the prevalence of appendicular skeletal muscle mass (ASM) in male patients with stable COPD. In addition, factors associated with parameters of ASM were also investigated.

We recruited 116 male patients with stable COPD from the outpatient clinic between September 2016 and December 2017. For each patient, we obtained demographic characteristics and measured post-bronchodilator forced expiratory volume in 1 second, symptoms, exacerbations history, and ASM. ASM was defined as the sum of the muscle masses of the 4 limbs.

Appendicular skeletal muscle mass index (ASMI) in male patients with stable COPD was 8.2 ± 0.9 kg/m^2^, and the prevalence of low skeletal muscle mass was 7.8% (9 of 116 patients). Multiple linear-regression analysis showed that body mass index, occupation, fat-free mass index, and the modified medical research council scale were significantly correlated with ASMI. Compared with nonexercise group, lower limb muscle mass and ASM were significantly improved in physical exercise group.

Underweight, retirement, fat-free mass depletion, and severe dyspnea are all risk factors for ASM in male patients with stable COPD. Our findings also justify the importance of exercise training in improving ASM.

## 1 Introduction

Chronic obstructive pulmonary disease (COPD) is a respiratory disease characterized by persistent airflow limitation and respiratory symptoms. COPD is a major global public health issue that may cause increased mortality, disability, and health care costs among individuals with this condition.^[1,2]^ There is emerging evidence that COPD is associated with various systemic disorders, including weight loss, nutritional abnormalities, and skeletal muscle dysfunction^[3]^ which is associated with inflammation, chronic hypoxia, oxidative stress reaction, protein synthesis, or decomposition imbalance.^[4]^

In COPD patients aged over 50 years of age, there is a reduction of 1% to 2% per year in muscle mass. Muscle wasting, a common phenomenon in COPD, is associated with a decline in peripheral muscle mass and strength.^[5]^ Appendicular skeletal muscle mass (ASM) was defined as the sum of the muscle mass of the 4 limbs, and appendicular skeletal mass index (ASMI) was calculated as ASM/height^2^ (ASM/m^2^) in the European Working Group for Sarcopenia guidelines.^[6]^ The prevalence of low ASM accompanied by poor physical performance was 14.5% among stable COPD patients.^[7]^ Limbs muscle wasting is a common disorder in COPD patients that limits daily activities and exercise intolerance, especially in males.^[8,9]^ Most studies have reported that prevalence and mortality of COPD are greater in men than in women.^[10,11]^ Therefore, it is important to determine the ASM of male patients with COPD.

A literature review found that skeletal muscle dysfunction was associated with worsening lung function, severity, and prognosis of COPD,^[12]^ which might contribute to systemic inflammation and insulin resistance. Thus far, the relationship between COPD severity and skeletal muscle mass has been investigated in Western^[4]^ or Southeast Asian patients,^[13]^ whereas no data was available for Chinese patients. Therefore, we aimed to estimate the prevalence of low ASM measured by bioelectrical impedance analysis (BIA) and to investigate the factors associated with ASMI in male patients with COPD.

## 2 Methods

### 2.1 Study design and participants

We conducted a cross-sectional study involving male patients aged older than 45 years with COPD treated at the outpatient clinic of the First Affiliated Hospital of Soochow University from September 2016 to December 2017. Participants were included if they had a diagnosis of stable COPD evaluated by spirometry with post-bronchodilator force expiratory volume in 1 second (FEV_1_) less than 70% of forced vital capacity, as defined by the Global Initiative for Chronic Obstructive Lung Disease (GOLD).^[14]^ We excluded subjects if they were participated in other studies or had a medical history or clinical condition known to interfere with body composition. A total of 183 patients were recruited; of 183 patients, 67 were excluded due to various reasons, including patients with acute respiratory symptoms (n = 36), denied participation (n = 18), and unable to complete the related questionnaires and tests (n = 13). Finally, 116 men agreed to participate in our study. The study was approved by the Ethics Committee of the First Affiliated Hospital of Soochow University, and all of the patients gave their informed consent before participating.

### 2.2 Assessments

#### 2.2.1 Body composition measurements

A multi-frequency BIA (BCA-2A; Tsinghua Tongfang, China) was used to measure body composition. The following body composition indicators were measured: height, body weight, body mass index (BMI), fat-free mass (FFM), upper limb muscle mass (a-SM), lower limb muscle mass (l-SM), and ASM. ASMI and fat-free mass index (FFMI) were expressed in ASM/height^2^ and FFM/height^2^, respectively. The following cutoff values were used to identify low muscle mass; ASMI of ≤7.0 kg/m^2^,^[15]^ and FFMI of ≤16 kg/m^2^.^[6]^ The patients were asked to avoid eating a meal 2 hours before the test. The participants in light clothing removed their shoes and stood on the platform. The measurement began when the participants placed their hands on the handgrips, while keeping arms straight and open at a 15-degree angle. Measurements were carried out by well-trained staffs, and completed within 2 minutes.

#### 2.2.2 COPD

The severity of COPD was assessed by 3 clinical parameters, including post-bronchodilator FEV_1_, symptoms, and exacerbations history.^[16]^ First, COPD evaluated by spirometry with post-bronchodilator FEV1 less than 70% of forced vital capacity, as defined by GOLD. The degree of airflow obstruction was classified into 4 grades: grade 1 (≥80%), 2 (50%–79%), 3 (30%–49%), and 4 (<30%). Second, the symptoms were assessed by the modified medical research council (MMRC) scale (0–1 or ≥2 points) and COPD assessment test (CAT) (≥10 or <10 points), with higher scores indicated more severe symptoms.^[17]^ Third, history of exacerbations was defined as the frequency of acute events characterized by a worsening of respiratory symptoms that lead to a change in medication in the past year (0–1 or ≥2 times). Lastly, the COPD severity was classified into 4 categories (A, B, C, D) according to GOLD.

#### 2.2.3 Physical exercise

In this study, we also investigate the influence of physical exercise on skeletal muscle mass. Physical exercise levels were calculated using the participants’ daily exercise. Any form of daily exercise performed less than once a week was defined as having no exercise. The subjects were divided into exercise group and nonexercise group, differences in skeletal muscle mass between the 2 groups were analyzed.

### 2.3 Statistical analysis

Normality was assessed using Kolmogorov–Smirnov tests. All data were presented as mean ± standard deviation. We used independent-sample *t* tests and 1-way analysis of variance for comparative analysis. In addition, we performed a stepwise multiple linear-regression analysis with ASMI as a dependent variable, and in that analysis we used only the variables that were significantly associated with ASMI in univariate analysis. All statistical analyses were performed using IBM SPSS 19.0. The level of statistical significance was set at *P* < .05.

## 3 Results

Table 1 shows the baseline characteristics of the 116 male patients with stable COPD. The average age was 66.6 ± 7.3 years. Average BMI was 22.4 ± 3.7 kg/m^2^, which was considered in normal-weight range. The average ASMI was 8.2 ± 0.9 kg/m^2^. Average FFMI was 17.6 ± 1.8 kg/m^2^. The prevalence of low skeletal muscle mass (ASMI ≤7.0 kg/m^2^) and FFM depletion (FFMI ≤16 kg/m^2^) were 7.8% (9 cases) and 19.8% (23 cases), respectively.

**Table 1:**
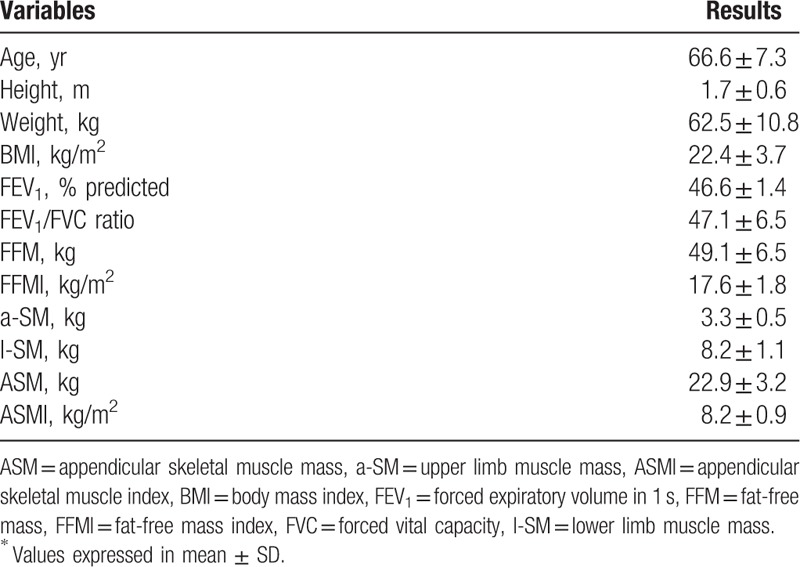
Baseline characteristics of the patients (N = 116)^∗^.

Age, occupation, BMI, and FFMI appeared to influence l-SM, ASM, and ASMI (all *P* < .05). Physical exercise group had significantly higher l-SM and ASM than nonexercise group (*P* < .05). There were no significant differences in duration and smoking status of COPD (*P* > .05) (Table 2).

**Table 2:**
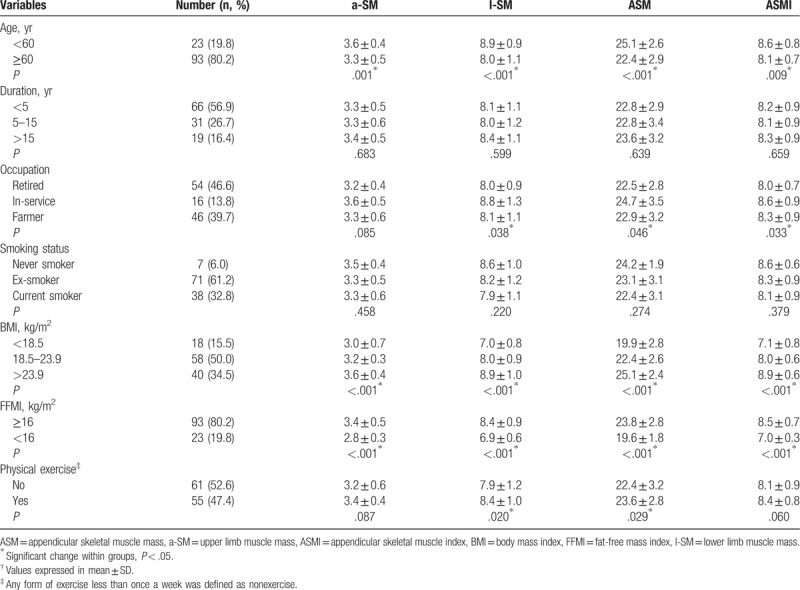
Parameters of ASM in male patients with COPD according to baseline characteristics^†^.

Parameters of ASM in male patients with COPD according to disease severity are shown in Table 3. Significant differences in ASMI were observed between FEV_1_% (*P* = .041), MMRC (*P* = .009) and CAT (*P* = .050) and GOLD categories groups (*P* = .049), except for exacerbation history (*P* = .258). MMRC was also associated with a-SM (*P < *.001), l-SM (*P* = .001), and ASM (*P < *.001).

**Table 3:**
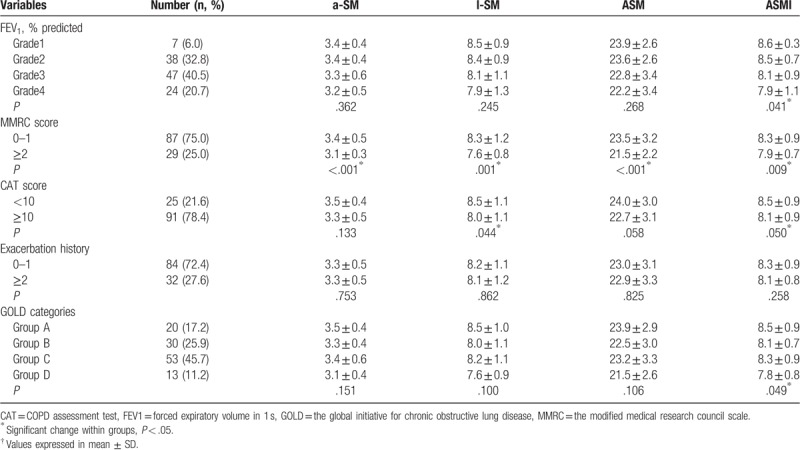
Parameters of ASM in male patients with COPD according to disease severity^†^.

The results of multiple stepwise linear-regression analysis are shown in Table 4. BMI, occupation, and FFMI were positively correlated with ASMI (*P* < .001 or *P* = .001, respectively), whereas MMRC was inversely correlated with ASMI (*P* = .002) (Table 5).

**Table 4:**
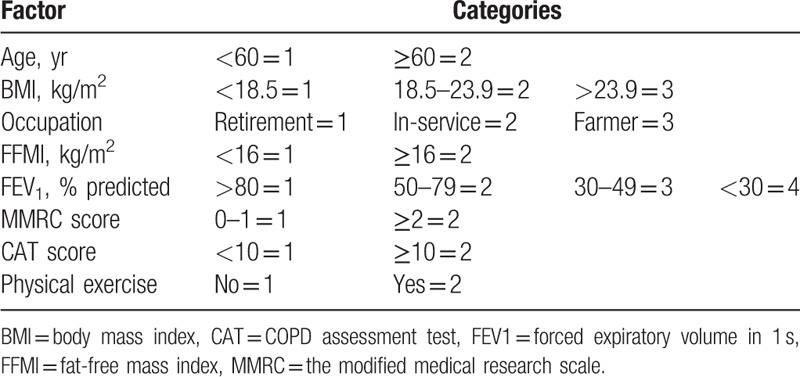
Factor category assignment table.

**Table 5:**
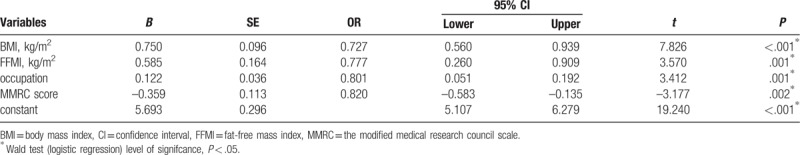
Multivariate analysis for factors influencing ASMI.

## 4 Discussion

In the present study, the prevalence of low skeletal muscle mass in male patients with COPD was lower than prior report in the Southeast Asian patients^[13]^ and British patients^[7]^ (7.8% vs 24% vs 14.5%, respectively). These findings could possibly be explained by the differences in measurement methods, diagnostic criteria, ethnicities, and regions. Unlike those studies,^[7,13]^ we measured the skeletal muscle mass using BIA rather than dual-energy X-ray absorptiometry, which was more easier to perform and portable for assessment of skeletal muscle mass in outpatient clinics according to the Asian working group for sarcopenia.^[15]^

The exact mechanisms underlying muscle wasting in COPD is poorly understood. Muscle wasting is one of the extrapulmonary complications of COPD.^[5]^ Chronic inflammation is thought to play a role in muscle wasting in COPD patients. In severe cases, chronic inflammation can alter muscle structure and its function by accelerating muscle catabolism and decreasing muscle strength.^[18]^ Furthermore, muscle wasting in COPD patients could be caused by various extrapulmonary disorders that often accompany this respiratory disorder, including malnutrition,^[19]^ systemic inflammation,^[20]^ and oxidative stress.^[21]^

We found that ASMI was associated with lung function and dyspnea assessed by MMRC dyspnea scale and CAT score, except for exacerbations. We also found that MMRC was an independent factor for ASMI. These findings indicated that the more advanced respiratory symptoms, the more reduction in skeletal muscle mass of the limbs. Dyspnea is a common reported symptom in COPD patients. The muscles involved in respiration have to work harder to improve dyspnea. In addition, breathing difficulty can lead the patients to involuntarily reduce their daily activity levels., Consequently, the patient may enter a feedback loop of “skeletal muscle consumption-exacerbation of respiratory symptoms-activity limitation.”^[22]^ While we found that skeletal muscle wasting was not significant in the early or mild condition of COPD, previous studies have indicated that COPD duration and acute exacerbation could accelerate muscle wasting.^[23–25]^ Altogether, it is essential for the COPD patients to control the disease and reduce exacerbations to prevent or at least delay muscle wasting.

Total body weight consists of fat mass and FFM (bones, organs, and muscle). Our findings indicated that BMI and FFMI appeared to influence ASMI. There were significant differences in a-SM, l-SM, ASM, ASMI, and ASM% among different BMI or FFMI groups (*P* < .05). Notably, patients with BMI <18.5 kg/m^2^ (underweight) or FFMI <16 kg/m^2^ (FFM depletion) had a significantly lower skeletal muscle mass than those in categories, suggesting that underweight and FFM depletion were associated with a lower skeletal muscle mass. By comparison, several studies showed that 20% to 30% of patients with COPD had a normal BMI, although a lower ASMI scores was also observed,^[26,27]^ indicating that BMI was a poor indicator for skeletal muscle mass in patients with COPD.^[28]^ Furthermore, ASMI was also more reliable indicator than BMI in determining the nutritional status of the patients.

Our results showed that occupation also associated with skeletal muscle mass, with retired participants had the lowest ASMI compared to other participants. The finding was reasonable because retired participants had less daily activity due to the change of lifestyle, which may eventually accelerate muscle dysfunction.^[29,30]^ Studies have shown that^[31]^ being sedentary may be associated with reduction in skeletal muscle mass, which leads to excess production of reactive oxygen species in skeletal muscle, aggravating of the activation of the redox response signaling pathway and the progression of muscle reduction. The results of this study showed that patients engaging in weekly exercise had significantly better l-SM and ASM than those who did not exercise (*P* < .05). Zhu Yaqiong found that the greater the muscle mass of the lower limbs was, the better the activity of the lower limb muscles.^[32]^ Several studies have confirmed^[33,34]^ that proper exercise training was beneficial to muscle physiology, as it may reduce inflammation, increase mitochondrial function, improve myogenic signals, and improve muscle mass and function. Therefore, exercise training is useful for disrupting the feedback loop of “skeletal muscle consumption – exacerbation of respiratory symptoms – activity limitation” in patients with COPD. In other words, we could use exercise training in the COPD pulmonary rehabilitation program to improve the skeletal muscle mass of patients.

### 4.1 Limitations

There were some limitations of the present study. First, the cross-sectional design only permits us to investigate associations. Therefore, the underlying cause-effect relationship cannot be determined. Prospective studies are warranted to confirm our findings. Second, given the present study only included a small number of patients treated in our hospital, our findings may not be generalized to other populations. Third, we only concentrated on ASM in male patients with COPD. It has been shown that low muscle mass was also evident in female patients.^[35]^ Third, factors associated with ASM may be influenced by COPD severity. Future studies should investigate whether ASM in COPD patients is influenced by important factors, such as sex, medications, severity of COPD, and COPD comorbidities.

## 5 Conclusions

Underweight, retirement, FFM depletion, and severe dyspnea are all risk factors for ASM in male patients with stable COPD. Our findings also justify the importance of exercise training in improving ASM.

## Acknowledgments

The authors thank all the participants and investigators at the Department of Respiratory Medicine, the First Affiliated Hospital of Soochow University. We are also grateful for the analyzers provided by the Department of Nutrition, the First Affiliated Hospital of Soochow University. The authors thank Khemayanto Hidayat for linguistic assistance during manuscript preparation.

## Author contributions

**Data curation:** Yi Chen, Yanan Kan, Min Geng.

**Formal analysis:** Meie Niu.

**Investigation:** Yanxia Han, Zhenyun Wu, Meie Niu.

**Methodology:** Hongying Qian, Meie Niu.

**Resources:** Yi Chen, Yanan Kan, Min Geng.

**Supervision:** Hongying Qian.

**Writing – original draft:** Yanxia Han.

**Writing – review and editing:** Nuo Xu, Hongying Qian, Haifang Wang, Meie Niu.
